# Supplementary material for: Low-Temperature Multiple Micro-Dispensing on Microneedles for Accurate Transcutaneous Smallpox Vaccination
Source: Vaccines (Basel). 2022 Apr 4;10(4):561. doi: 10.3390/vaccines10040561 (PMC9024753; doi:10.3390/vaccines10040561)
Supplement: Supplementary file 1 [file vaccines-10-00561-s001.zip › vaccines-1637809-supplementary.pdf]

Supplementary materials

Low-temperature multiple micro-dispensing on microneedles for accurate transcutaneous smallpox vaccination

Sang-Gu Yim [1,†], Yun-Ho Hwang [2,†], Seonyeong An [1], Keum-Yong Seong [1], Seo Yeon Kim [2], Semin Kim [3], Hyeseon Lee [1], Kang-Oh Lee [3], Mi Young Kim [2], Dokeun Kim [2], You-Jin Kim [2,*] and Seung Yun Yang [1,*]

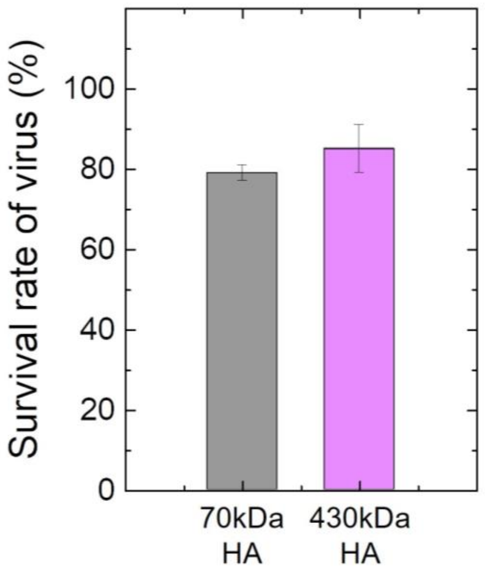

Figure S1. Effect of the MW of HA used as a viscous enhancer on the activity of smallpox vaccine (vaccinia virus) in coating solutions.

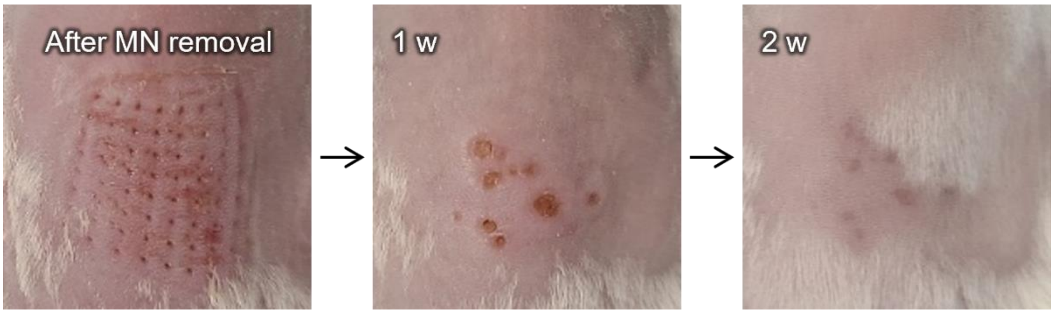

Figure S2. Photographs showing the cutaneous reaction at the inoculation site with vaccinia virus-coated MN patch.

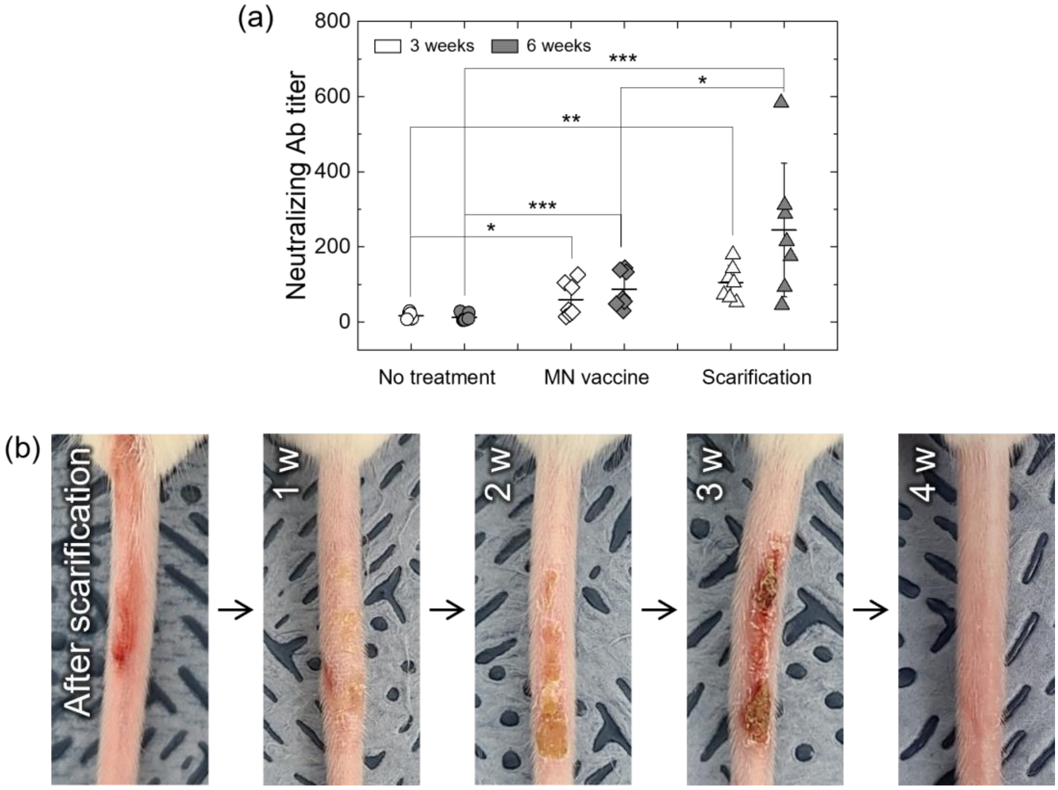

Figure S3. (a) Neutralizing antibody titers at 3 and 6 weeks after immunization with smallpox vaccine (2×10^5 PFU) using tail scarification technique. For immunization, female BALB/C mice were vaccinated by applying a droplet of 20 μL of vaccine solution on the dorsal base of the tail followed by scratching the skin with an 18G needle. Serum samples of mice were collected at 3 and 6 weeks after vaccination (n=7). (b) Photographs showing the cutaneous reaction at the vaccination site inoculated by scarification. Notably, a more severe cutaneous reaction was observed after inoculation by scarification, and the scar remained at 4 weeks. Statistical significance compared to the no treatment sample was determined by a t-test (*$p < 0.05$, **$p < 0.01$, ***$p<0.005$).
